# Novel biomechanical injury risk score demonstrates correlation with lower limb posterior chain injury in 50 elite-level rugby union athletes

**DOI:** 10.1136/bmjsem-2021-001062

**Published:** 2021-10-19

**Authors:** Rhys Hughes, Matt Cross, Keith Stokes, Daniel Tobin, Eoin Power, Steph McNally, Jonathan Pamment

**Affiliations:** 1 Performance Medical Department, Gloucester Rugby Ltd, Gloucester, UK; 2 Department for Health, University of Bath, Bath, UK; 3 Research and Rugby Development, Premier Rugby Ltd, Twickenham, UK; 4 Medical Research, Rugby Football Union, Twickenham, London, UK

**Keywords:** sports physiotherapy, sports & exercise medicine, biomechanics, hamstring, pelvis

## Abstract

**Objectives:**

Lower limb posterior chain injury (PCI) is common among athletic populations, with multifactorial risk factors including age, previous injury, strength measurements, range of motion and training load. Biomechanics are commonly considered in the prevention and rehabilitation of PCI by performance staff. However, there is no documented testing method to assess for associations between biomechanics and PCI. The aim of this study was to investigate whether there is an association between an easily applicable, novel biomechanical assessment tool and PCI.

**Methods:**

Fifty male elite-level rugby union athletes (age 22.83±5.08) participating in the highest tier of England were tested at the start of the 2019 preseason period and PCIs (N=48) were recorded over the 2019/2020 playing season. Participants’ biomechanics were analysed using two-dimensional video analysis against an injury risk score (IRS) system in the performance of the combined movement—prone hip extension and knee flexion. Participants’ biomechanics in carrying out this movement were scored against the 10-point IRS, where the more compensatory movement recorded sees an increase in an individual’s IRS. Participants’ IRS was then compared against the number of PCIs sustained and Spearman’s correlation coefficient was used for statistical analysis.

**Results:**

There is a significant association between IRS and PCI (R=0.542, p<0.001). Linear regression demonstrated that an increase in 1 in IRS was associated with a 35% increase in PCI incidence (R²=0.346).

**Conclusion:**

A significance between the IRS and PCI provides preliminary support for its use as an injury risk assessment tool.

**Key messages**

**What is already known**

- Lower limb posterior chain injury including the lower back, hamstring and calf complex is considered multifactorial with both modifiable and non-modifiable risk factors.
- Recently, a relationship between hamstring injury and biomechanics has been demonstrated but whether this is the cause or effect of injury is unknown.
- There is currently no uniformed assessment method for lower limb biomechanics in posterior chain injury.

**What are the new findings**

- The novel biomechanical injury risk score can be used to quantify an individual’s risk of lower limb posterior chain injury, providing a uniformed assessment method that is easily applied within the constraints of a sports performance-medical environment.
- While mitigating posterior chain injury risk, the assessment also provides practitioners target areas for biomechanical intervention in the management and prevention of posterior chain injuries.
- It is proposed that the injury risk score forms part of a test cluster alongside an individual’s demographics, training load monitoring, strength and range of motion assessment, to ensure a comprehensive analysis in the management of posterior chain injury risk.

## Introduction

The term posterior chain injury (PCI) is commonly used in relation to injuries to the posterior musculoskeletal system of the lower limb.^1 2^ PCI is commonly observed within athletic populations, with a reported time loss of 10 hours for every 1000 playing hours within elite-level sport.^3^ The most prevalent PCIs involve structures within the posterior musculoskeletal system such as the trunk, pelvis, hamstring and calf complexes.^4–8^ This has subsequently led to a large amount of research being conducted that focuses on these areas of PCI, particularly during high-velocity activities such as sprinting. Sprinting requires multiple structures of the posterior musculoskeletal system to work concurrently.^8 9^ Occurrence of PCI is attributed to a failure and a loss of concurrence somewhere within this system.^9 10^ Therefore, it is no surprise that PCI prevalence is high in many sports that require sprinting, including rugby union,^3^ soccer,^11^ American football^12^ and athletics.^13^ Of the structures within the posterior musculoskeletal system, the hamstring complex is most frequently affected.^14^

Research in athletic populations has found the non-modifiable risk factors of: (1) greater age and (2) previous injury to be predictors of PCI.^15 16^ Modifiable risk factors suggested to influence PCI include deficits in strength, particularly eccentric strength in hamstring injury^17^; overload in training volume^18 19^; sprint performance^20^ and reduced ranges of motion.^21^ The most influential finding regarding modifiable risk factors relating to PCI management has arguably been the introduction of the Nordic hamstring extension in 2001.^4 17 22–24^ Many exercise programmes focusing on the prevention or rehabilitation of PCI aim to influence these modifiable factors and include some form of coached lower limb biomechanical positioning during exercise prescription.^25^ However, there is a lack of evidence of the effect biomechanics may have on PCI specifically. While pelvic and lower limb positioning is assessed in many ways by practitioners in sport performance-medical settings, until recently,^20 26 27^ there has been no evidence of a relationship between biomechanics and PCI. It is also worth noting that there is currently no uniform clinical assessment method of pelvic or lower limb mechanics that relates to PCI.

A multimodal intervention of bed-based treatment, mobility and exercise therapy has demonstrated positive influence on pelvic biomechanics when assessed using an inertial sensor.^26^ The authors suggest a neutral pelvis position and relative limb alignment could reduce hamstring strain injury risk; however, there is a lack of uniform assessment and comparison to injury incidences to conclude this. Further research^20^ also supports a link between these factors highlighting athletes who have sustained a PCI present with an increased amount of anterior pelvic tilt through the gait cycle of running when compared against those who do not. Yet, no conclusion was drawn as to whether this was the cause or effect of the injuries and no evidential comparison can be made. The role of muscle cocontraction in biomechanics when analysing electromyography (EMG) patterns within soccer players has also been investigated.^27^ Athletes who demonstrated an inefficient cocontraction pattern when recruiting specific stabilising muscles within the posterior musculoskeletal system (lumbar spine erectors, gluteus maximus and hamstrings) were up to 8 times more susceptible to PCI.^27^ It is evident that association between an individuals’ biomechanics and PCI risk may be present but need further evidential support with direction on a uniform assessment method.

This study assesses the association between an easily applicable, novel, clinician-assessed biomechanical injury risk score (IRS) and the number of PCI among a group of elite-level rugby union athletes.

## Methods

### Study participants

Fifty adult male professional rugby union athletes aged between 18 and 35 years (22.8±5.1) were recruited at the start of a full playing season (First of July 2019) and were considered relatively well rested after a minimum of 5 weeks break between playing seasons. The participants were injury free and considered available for full training at the time of testing.

### Study design

This study investigates the association between a clinician assessed IRS system and PCI sustained during one full playing season. Due to the COVID-19 pandemic shortening the season, deviation from the original protocol occurred, so PCI data were collected across the first 9 months of the season instead of the originally planned 11 months. PCI was defined as any non-contact injury to the foot (palmar aspect), calf complex (including the achilles tendon), adductor muscle group, hamstring muscle group, gluteal muscle group, lumbar spine and thoracic spine regions that led to time loss in training and/or matches. PCI included soft tissue disruption categorised using the British Athletics Muscle Injury Classification,^28^ which details tissue disruption of all types between grades 0 and 4; discopathy, neuropathy, tendonopathy, and overload injuries were also included. Contact injuries from direct impact were excluded from the study as direct impact injuries are not generally deemed to be affected by biomechanics.

### The IRS

**Figure 1:**
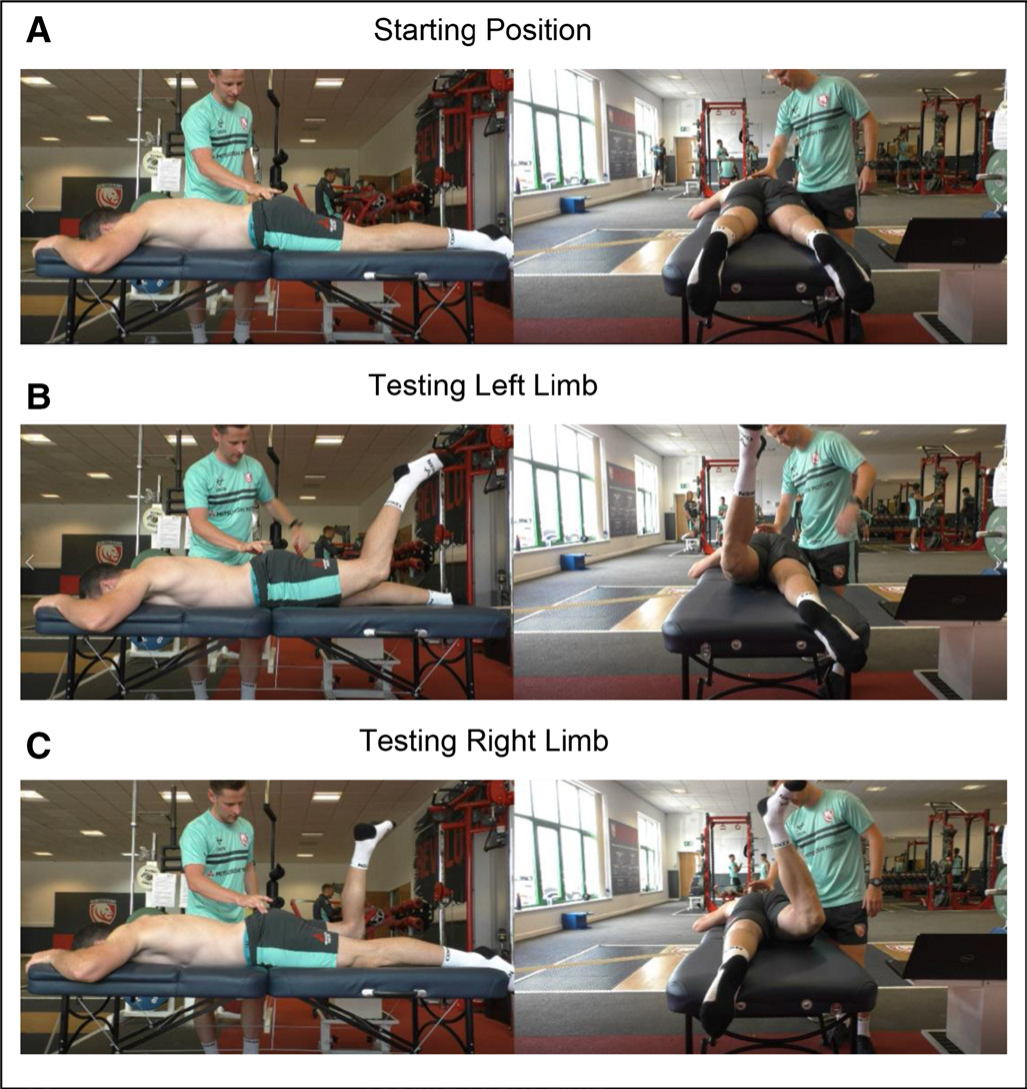
Still image views of longitudinal (left) and transverse (right) views of biomechanical deficit and limb asymmetry when using the IRS system: starting position (A), testing left limb (B) and testing right limb (C).

**Figure 2:**
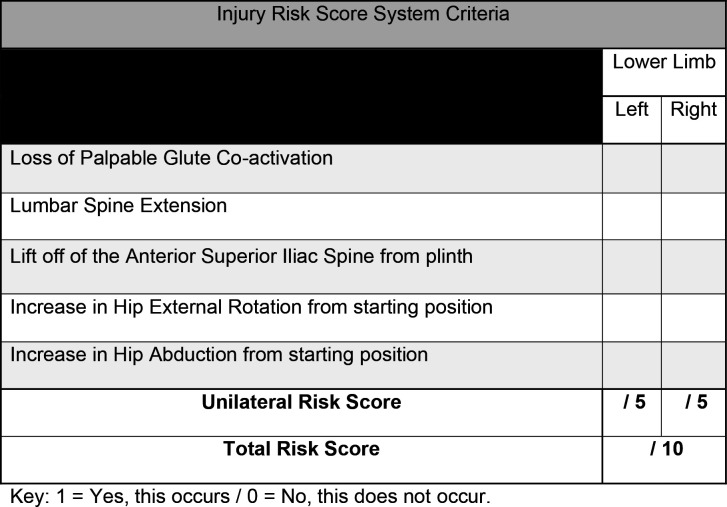
IRS system criteria.

A visual representation of the testing procedure is presented in figure 1 and an example of the IRS system criteria in figure 2. This novel testing protocol is clinician assessed and it is not documented in the empirical literature. However, the assessment method uses a modified approach that used EMG in assessing prone hip extension (PHE), which demonstrated good levels of reliability and validity.^27^ The modification translated into this study sees the replacement of EMG with palpable assessment of gluteal muscle tone, the addition of prone knee flexion (PKF) and observation of biomechanics retrospectively analysed using two-dimensional video analysis. Biomechanics are assessed against the IRS system in the performance of combined PHE and PKF (figure 1), subsequently providing greater comparison to the gait cycle than PHE alone, where PHE and PKF are performed concurrently.^20^ Participants can score a maximum of 10 points following the IRS system criteria—the more biomechanical change observed from the participants anatomical prone starting position, the higher the score. The five compensatory criteria include: (1) loss of palpable coactivation in either gluteal muscle, (2) lumbar spine extension, (3) lift of the anterior superior iliac spine from the plinth, (4) an increase in hip external rotation (5) an increase in hip abduction. If the athlete displays a compensatory movement pattern for one of the five criteria, 1 point would be scored per limb, left (maximum 5 points) and right (maximum 5points) with a maximum of 10 points achievable.

As the IRS system is novel, a repeatability pilot was performed prior to the data collection and results were analysed using Fleiss Kappa to assess inter-rater reliability (IRR). The lead researcher educated and familiarised four assessors in the IRS system. Each clinician had a minimum of 5 years experience in musculoskeletal medicine. The clinicians were blinded and asked to analyse a small pilot group (four participants), against the IRS system. Results from this pilot study were interpreted as suggested by McHugh,^29^ whereby (R≥0.90—excellent, R=0.80–0.89—very good, R=0.60–0.79—good, R=0.40–0.59—moderate and R=0.21–0.39—minimal). Completing this pretrial pilot demonstrated good levels of IRR (R=0.651, p<0.001) and allowed for some minor adjustments in the logistics around participant testing, reflected in the testing procedure section.

### Testing procedure

Each participant started in a prone position on a treatment plinth with arms placed by their side or above their head (as in figure 1) to negate the use of upper limb stabilisation during testing. The testing clinician then placed their index finger and thumb of one hand on the horizontal plateau of the gluteus maximus to assess palpable gluteal activation.^30^ Prior to testing, each participant was read the same script by the testing clinician, *Tense both buttock muscles and maintain this contraction for the duration of the movement, until told the test is over. Now bend your left knee to 90 degrees and lift that knee off the bed*. The testing practitioner would then say, *The test is over*. The participant then relaxes, resuming the prone starting position. The opposite side would then be tested with the same instructions where *left knee* was replaced with *right knee*. Each participant was given a 2 min testing window and participants were tested consecutively. The testing clinician retrospectively assessed each participant through the combined PHE and PKF movement against the IRS criteria, using video analysis. The only live score recorded during the testing protocol was palpable gluteal activation as muscle tone was deemed unrecordable by video analysis. All scores were recorded on a laptop using spreadsheet software.

### Setting and equipment

Equipment included: a password-protected external hard drive; computer compatible with spreadsheet software and the SPSS V.25^31^ for analysis; height-adjustable treatment plinth (plinth height was maintained throughout); three high-resolution video-recording cameras (Panasonic HC-V770 50× Zoom 4k Full HD) with tripods, capable of recording in two dimensions at perpendicular angles, where the horizontal centre line of the camera was lined up with the horizontal surface of the treatment plinth. The plinth height from the surface to the floor was set to 75 cm. Cameras were set up at 150 cm away from the edge of the treatment plinth to the lens. Video cameras were synchronised to record simultaneously from two angles at the start of testing. Recording angles included a lateral view of both left and right sides of the participants and a caudal to cephalad view. Footage was cropped to allow for time efficiency when assessing IRS by deleting plinth transfer footage between participants using video analysis software.^32^ This method of video augmentation in movement analysis is well validated^33 34^ and has proven reliable when qualitatively assessing human biomechanics against quantitative criteria.^35–37^

### Data collection

Participant’s IRS data were collected by a senior team physiotherapist and participant’s PCI data were collected over the course of the season by an independent member of the team’s therapy staff. Data collection for IRS took place within the physiotherapy room of an English premiership rugby union club. Participants were required to spend a maximum of 2 min each (including transfer time) in a prone position on the treatment plinth. Testing for all participants took approximately 2 hours including equipment setup. Data were collected from all participants on day 1 of preseason testing. The number and location of PCIs for each participant were then recorded for the duration of the 9-month English premiership rugby playing season using digital spreadsheet software as part of departmental injury surveillance. This was standardised using the definition of PCI as outlined in the study design, and the same individual was responsible for data recording to ensure continuity and avoid potential for bias.

### Outcome measures

The two main outcome measures for this study were PCI (dependent variable) and the IRS (independent variable). The IRS was measured quantitatively from analysis of the qualitative data collected against the IRS and cross-referenced against the recorded video by the lead researcher. The number of PCIs sustained by each player across a 9 month playing season was captured at the end of the season. Participant age was also compared against the number of PCIs sustained for use in data comparison against the already established injury risk identifier.

### Statistical analysis

Data analysis using SPSS version 25^31^ was used for data comparisons and assessment of correlation. Prior to statistical analysis both IRS and PCI variables were assessed for homogeneity of variance and normal distribution using the Kolmogorov-Smirov and Shapiro-Wilk tests. As assumptions of homogeneity and normal distribution were not met, a Spearman’s correlation coefficient (SCC) was conducted where R was interpreted as (R≥0.90—very strong, R=0.70–0.89—strong, R=0.40–0.69—good, R=0.10–0.39—weak and R=0–0.09—negligible).^38^ As age is proven to be a significant predictor of injury, it was considered a confounder; hence, appropriate adjustment was made to control this factor in the statistical analysis.

To reduce the likelihood of any type 1 errors within the group (N=50), a critical alpha (p) of <0.05 was used. Finally, a non-parametric linear regression was conducted to demonstrate whether there was a linear relationship between PCI and IRS, and if so to what degree the variables were related. To ensure methodological rigour throughout the study adheres to STROBE (Strengthening The Reporting of OBservational Studies in Epidemiology)^39^ reporting guidelines.

### Patient and public involvement

Participants were first involved in research during the first day of data collection when informed consent was obtained, and their IRS was recorded. The research question and outcome measures were developed by the authors, and participants were informed of these using a patient information sheet prior to data collection. Participants were not involved in study design, recruitment or conduction and they were not asked to assess the burden or time required to participate in the study. In the dissemination of the results, the participants received their individual IRS followed by a discussion around their PCI risk with advice around reducing this risk.

## Results

**Figure 3:**
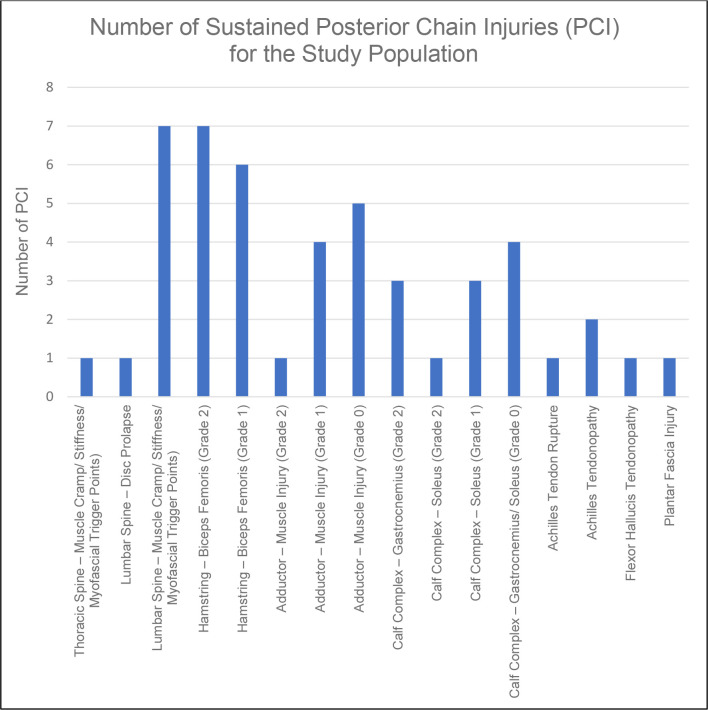
Number of posterior chain injuries (PCI) for the study population.

Fifty subjects participated in the study (age 22.83±5.08). The mean IRS score across the population was 5.80±1.74 and participants IRS ranged from 3 to 10 with a median of 7. A total of 48 PCIs were observed in 30 of the participants across the playing season, as displayed in figure 3. Twenty participants did not suffer a PCI, 18 suffered 1 PCI, 7 suffered 2 PCIs, 4 suffered 3 PCIs and 1 suffered 4 PCIs. The average number of PCIs across the population was 0.90±1.03 per participant. The most frequently observed PCIs were lumbar spine—(muscle cramp/stiffness/myofascial trigger points) and hamstring—biceps femoris (grade 2) both recording seven incidences across the season (figure 3). The number of biomechanical compensations observed across the group was 320, with some common themes in compensation, as displayed in table 1.

**Table 1:** IRS system points scored for each biomechanical compensation.

	Loss of palpable glute co-activation	Lumbar spine extension	Lift off of the anterior superior iliac spine from plinth	Increase in hip external rotation from starting position	Increase in hip abduction from starting position	Total
Total points for each biomechanical characteristic	62	95	71	54	38	**320**
Percentage	19	30	22	17	12	

Additionally, when the IRS was compared between participants who suffered a PCI (30) against those who did not (20) the following was observed, PCI sufferers mean IRS was 6.8±1.62 and non-PCI sufferers mean IRS was 5.4±1.43.

IRS, injury risk score.; PCI, posterior chain injury.

**Figure 4:**
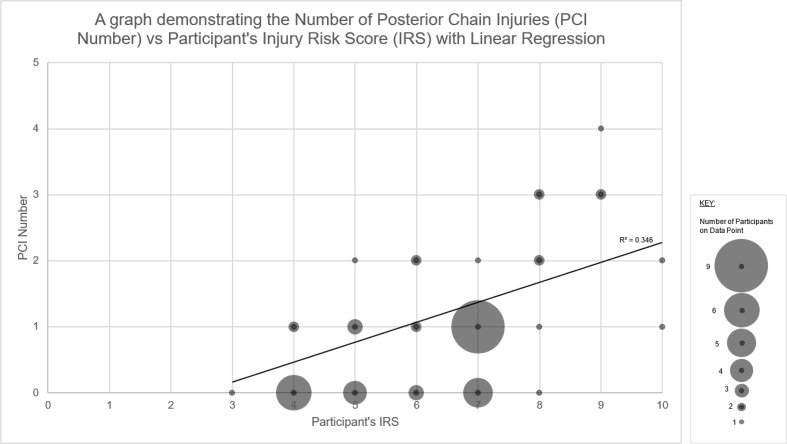
A graph demonstrating the number of posterior chain injuries (PCI number) versus injury risk score (IRS) with linear regression.

Spearman’s correlation demonstrated a good positive association between participants’ IRS and the number of PCIs while controlling for participant age (R=0.524 (p<0.001)) (figure 4). Linear regression identified that for every 1-point increase in IRS, individuals were 35% more likely to sustain a further PCI (R²=0.346) (figure 4).

## Discussion

With significance demonstrating association between the IRS and PCI, an increase in the identified biomechanical compensations is suggestive of increasing an individual’s risk of PCI. These results also provide confidence in the IRS as a biomechanical assessment tool for injury risk of PCI and provide guidance on biomechanical areas for intervention. Hence, practitioners may use the IRS alongside other pre-existing methods of identifying PCI risk, while guiding injury prevention. The IRS also has capacity to provide practitioners with areas for biomechanical improvement in the prevention of PCI when considering table 1. These suggest target areas for reducing an individual’s IRS when considering reduction of PCI risk. For example, lumbar spine extension is highlighted as the most common compensatory movement in the IRS across the population. With levels of significance in the association between PCI and the IRS assessment tool, it could be suggested that reducing lumbar spine extension which in turn lowers an individual’s IRS, could reduce the PCI risk. This could also be suggested for the other biomechanical areas of the IRS, although further investigation would be required to prove this.

### Limitations

Despite the injury surveillance data originally being planned across a full season, it is worth noting that data collection was reduced to 9 months due to the COVID-19 pandemic in March 2020 with approximately 25% of the season’s games remaining. Injury surveillance data are missing the last 2 months of the season when injuries may be most prevalent, possibly limiting true findings.^3^ Further limitation is also present when looking at the timing of data collection. The study design analyses biomechanics at a single point in time (preseason IRS testing). This is then compared with PCI occurring at multiple points through the season, some months apart and there is no evidence as to whether an individual’s IRS may have changed during this time prior to injury.

Additionally, the IRS quantifies an individual score for each participant based on a subjective analysis of movement, which will always be open to interpretation as with any descriptive method of movement analysis.^40^ This enforces the need for careful undertaking of the methodology in any replication of this study. A new test should demonstrate appropriate levels of reliability and validity and while the methodology gives clear direction in performing the IRS system with good pilot levels of IRR, this is a novel test, and the reliability levels are not deemed statistically very good or excellent. Therefore, when making conclusions on the IRS as a valid tool for assessing injury risk, this should be considered. Further studies could improve the reliability and strength of the IRS in completing a larger IRR analysis. In addition, while the inclusion criteria required individuals to be injury free and well rested at the time of IRS testing, no adjustment was made for previous injury history. Among athletic populations, it is difficult to find athletes without previous injury history, particularly at elite level but as one of the largest risk factors to injury, this could be considered a further limitation.

### Future studies

Further work into the comparison between the IRS and PCI would be beneficial among a larger sample size to improve levels of confidence between variables. It may also be of interest to first compare the IRS against an established injury predictor such as strength assessment.^23^ Any correlation between an established injury risk predictor and IRS system would further strengthen its use as an assessment tool. Correlation between the IRS and PCI could also be suggestive of a relationship between the prone test and upright running. Performing PHE and PKF is a key component of human movement, particularly during running gait.^16 27 41 42^ As the correlation in prone testing to upright running positions are unknown, formal comparison is required. An upright testing position may provide greater functional comparison although may present difficulties when standardising test protocol.

## Conclusion

The results demonstrate significant correlation between the novel IRS system compared with the number of PCIs within an athletic population. This study adds evidence that demonstrates that an increased IRS has an effect on an individual’s PCI risk and provides guidance for a uniformed assessment tool in assessing this risk. Establishing individual IRS for athletes in this way may aid in preventing and managing PCI that leads to time loss in training and matches. The IRS system can be used easily within musculoskeletal settings requiring minimal equipment, making it easily applicable around the constraints of performance-medical environments.

## Data Availability

Data are available upon reasonable request. Deidentified participant data may be available upon reasonable request.
